# Race and Gender Differences in Anterior Cruciate Ligament Femoral Footprint Location and Orientation: A 3D‐MRI Study

**DOI:** 10.1111/os.13918

**Published:** 2023-11-12

**Authors:** Lihang Zhang, Tianwen Huang, Changzhao Li, Xing Xing, Diyang Zou, Dimitris Dimitriou, Tsung‐Yuan Tsai, Pingyue Li

**Affiliations:** ^1^ Guangdong Key Lab of Orthopedic Technology and Implant, General Hospital of Southern Theater Command of PLA, The First School of Clinical Medicine Southern Medical University Guangzhou China; ^2^ Department of Joint Surgery The First Affiliated Hospital of Sun Yat‐sen University Guangzhou China; ^3^ Department of Biostatistics Bioinformatics & Biomathematics Georgetown University Washington, DC USA; ^4^ School of Biomedical Engineering & Med‐X Research Institute Shanghai Jiao Tong University Shanghai China; ^5^ Engineering Research Center of Digital Medicine and Clinical Translation Ministry of Education Shanghai China; ^6^ Department of Orthopedic Surgery, Shanghai Key Laboratory of Orthopedic Implants and Clinical Translational R&D Center of 3D Printing Technology, Shanghai Ninth People's Hospital Shanghai Jiao Tong University School of Medicine Shanghai China; ^7^ Department of Orthopedics University Hospital Balgrist Zurich Switzerland

**Keywords:** Anatomical single‐bundle reconstruction, Anterior cruciate ligament, Ethnicity, Femoral footprints, Gender, Orientation of ligament

## Abstract

**Objective:**

The femoral tunnel position is crucial to anatomic single‐bundle anterior cruciate ligament (ACL) reconstruction, but the ideal femoral footprint position are mostly based on small‐sized cadaveric studies and elderly patients with a single ethnic background. This study aimed to identify potential race‐ or gender‐specific differences in the ACL femoral footprint location and ACL orientation, determine the correlation between the ACL orientation and the femoral footprint location.

**Methods:**

Magnetic resonance images (MRIs) of 90 Caucasian participants and 90 matched Chinese subjects were used for reconstruction of three‐dimensional (3D) femur and tibial models. ACL footprints were sketched by several experienced orthopedic surgeons on the MRI photographs. The anatomical coordinate system was applied to reflect the ACL footprint location and orientation of scanned samples. The femoral ACL footprint locations were represented by their distance from the origin in the anteroposterior (A/P) and distal‐proximal (D/P) directions. The orientation of the ACL was described with the sagittal, coronal and transverse deviation angles. The ACL orientation and femoral footprint position were compared by the two‐sided *t*‐test. Multiple regression analysis was used to study the correlation between the orientation and femoral footprint position.

**Results:**

The average femur footprint A/P position was −6.6 ± 1.6 mm in the Chinese group and −5.1 ± 2.3 mm in the Caucasian group, (*p* < 0.001). The average femur footprint D/P position was −2.8 ± 2.4 mm in Chinese and − 3.9 ± 2.0 mm in Caucasians, (*p* = 0.001). The Chinese group had a mean difference of a 1.5 mm (6.1%) more posterior and 1.1 mm (5.3%) more proximal in the position from the flexion‐extension axis (FEA). And the males have a sagittal plane elevation about 4–5° higher than females in both racial groups. Furthermore, for every 1% (0.40 mm) increase in A/P and D/P values, the sagittal angle decreased by about 0.12° and 0.24°, respectively; the coronal angle decreased by about 0.10° and 0.30°, respectively. For every 1% (0.40 mm) increase in D/P value, the transverse angle increased by about 0.14°.

**Conclusion:**

The significant race‐ and gender‐specific differences in the femoral footprint and orientation of the ACL should be taken in consideration during anatomic single‐bundle ACL reconstruction. Furthermore, the quantitative relationship between the ACL orientation and the footprint location might provide some reference for surgeons to develop a surgical strategy in ACL single‐bundle reconstruction and revision.

## Graphical abstract

There were significant racial and gender differences in anterior cruciate ligament (ACL) femoral footprint and orientation. It was hoped that the quantitative relationship between the ACL femoral footprint location and orientation could provide some reference for surgeons during anatomic single‐bundle ACL reconstruction.

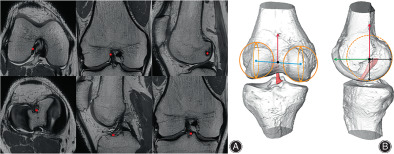

## Introduction

The anterior cruciate ligament (ACL) is the most vulnerable ligament of the knees to injury.^1^, ^2^, ^3^, ^4^ As more adolescents participate in high‐level sports and older adults remain active for longer periods of time, the incidence of ACL injury and reconstruction may be higher than previously reported.^1^ After ACL reconstruction, the injured knees still have varying degrees of dysfunction and enter the degenerative stage in advance.^5^, ^6^, ^7^, ^8^

The main concept of anatomic ACL reconstruction is to structurally and functionally restore the native ACL attachment, and thereby achieving better clinical results than non‐anatomic ACL reconstruction.^9^, ^10^ Accurate tunnel placement requires an excellent knowledge of the ACL footprint. Although several articles have described the location of femoral footprints, most were based on small‐sized cadaveric studies involving the elderly, unpaired subjects of a single ethnic background, and using two‐dimensional techniques.^11^, ^12^, ^13^ And potential differences in the femoral ACL footprint location and orientation between different races and genders have not been identified in the previous studies. Furthermore, perfect anatomical reconstruction usually does not result in intercondylar notch impingement in the extension position of the knee,^14^, ^15^, ^16^ however, it was still reported that the position of the femoral tunnel in a considerable number of patients had different degrees of deviation from the anatomical center of the femoral footprint (especially in the cases of ACL revision and chronic ACL injury).^17^, ^18^, ^19^ This may lead to intercondylar impingement and thus rupture of the graft. Therefore, analyzing the quantitative relationship between the ACL femoral footprint position and the ACL direction might help the surgeon to adjust the internal opening position of the femoral tunnel according to the intraoperative X‐ray images, quantitatively change the direction of the graft, and thus provide a certain auxiliary reference for avoiding the intercondylar notch impingement.

Since several epidemiological and anatomical studies have reported that ACL injury rates vary by sport, gender, and type of exposure, it is important to recognize these differences when assessing the effectiveness of evidence‐based, targeted prevention efforts.^20^, ^21^, ^22^, ^23^, ^24^, ^25^ Therefore, we speculated that the ACL femoral footprint in Chinese would differ significantly from that in Caucasians. Thus, the purposes of the present study were: (i) to explore the three‐dimensional, morphological differences in the femoral ACL footprint and ACL orientation between Chinese and Caucasian individuals with intact ACLs; and (ii) to determine the correlation between the ACL orientation and location of the femoral ACL footprint.

## Materials and Methods

### Patient Selection and Study Design

Medical records and magnetic resonance imaging (MRI) databases were reviewed after obtaining informed consent from all the patients and the ethics committee of Chinese PLA Southern Theater Command General Hospital [IRB 2019 No.10]. We also established a unified set of MRI protocols with our partner units (Department of Orthopedics, University Hospital Balgrist, Zurich, Switzerland) (Ethics Committee Northeast and Central Switzerland 2018‐01410). Inclusion criteria included patients aged less than 45 years of age who had no diagnosed knee problems. A retrospective analysis of 647 Caucasian patients from January 2015 to December 2017 and 732 ACL‐intact Chinese from October 2019 to December 2022 was performed. Our patient information database clearly records the age, gender, height, weight and body mass index (BMI) of all the patients. When we compared the above information for all patients, we found that 93 Caucasians (60 males and 33 females) and 102 Chinese (72 males and 30 females) people matched each other (60 *vs*. 72 males, 33 *vs*. 30 females). In order to pair the patients, after randomized elimination of 12 Chinese males and three Caucasian females, a cohort of 60 men and 30 women was formed (Table 1) (all *p* > 0.05). A review of MRI images was performed by three senior orthopedists (Li, Chen, and Shen) to screen for associated injuries. Exclusion criteria included meniscus tears, significant knee joint degeneration, and the inability of MRI images to be used to identify or reconstruct ACL footprints due to poor quality.

**TABLE 1:** Patient characteristics

Parameter	Chinese (*n* = 90)			Caucasian (*n* = 90)			*p* value and statistic value (*t*)					
	Male (*n* = 60)	Female (*n* = 30)	Total (*n* = 90)	Male (*n* = 60)	Female (*n* = 30)	Total (*n* = 90)	t1	P1	t2	P2	t3	P3
Age (year)	24.9 (21–43)	26.2 (17–39)	25.3 (17–43)	25.3 (21–43)	25.0 (17–40)	25.2 (17–43)	−0.427	0.656	1.289	0.210	0.037	0.802
Weight (kg)	72.4 (58.1–88.7)	58.6 (43.2–73.1)	67.8 (43.2–88.7)	75.3 (60.3–91.9)	62.8 (46.3–81.3)	71.1 (46.3–91.9)	−0.965	0.229	−1.425	0.115	−1.333	0.183
Height (cm)	175.3 (166–184)	164.6 (155–173)	171.7 (155–184)	177.7 (169–187)	166.5 (160–175)	174.1 (160–187)	−1.876	0.075	−1.541	0.129	−1.881	0.07
BMI (kg/m^2^)	23.5 (20.4–27.6)	22.5 (19.6–26.8)	23.2 (19.6–27.6)	24.3 (20.7–28.3)	22.9 (19.7–27.3)	23.8 (19.7–28.3)	−0.543	0.567	−0.356	0.834	−0.356	0.834

*Notes*: No significant difference was identified in age, weight, height and BMI between the Chinese male and Caucasian male groups (p1), Chinese female and Caucasian female groups (p2), and Chinese and Caucasian groups (p3). Statistic values (t1, t2, t3).

### Magnetic Resonance Parameters and Protocols

**FIGURE 1:**
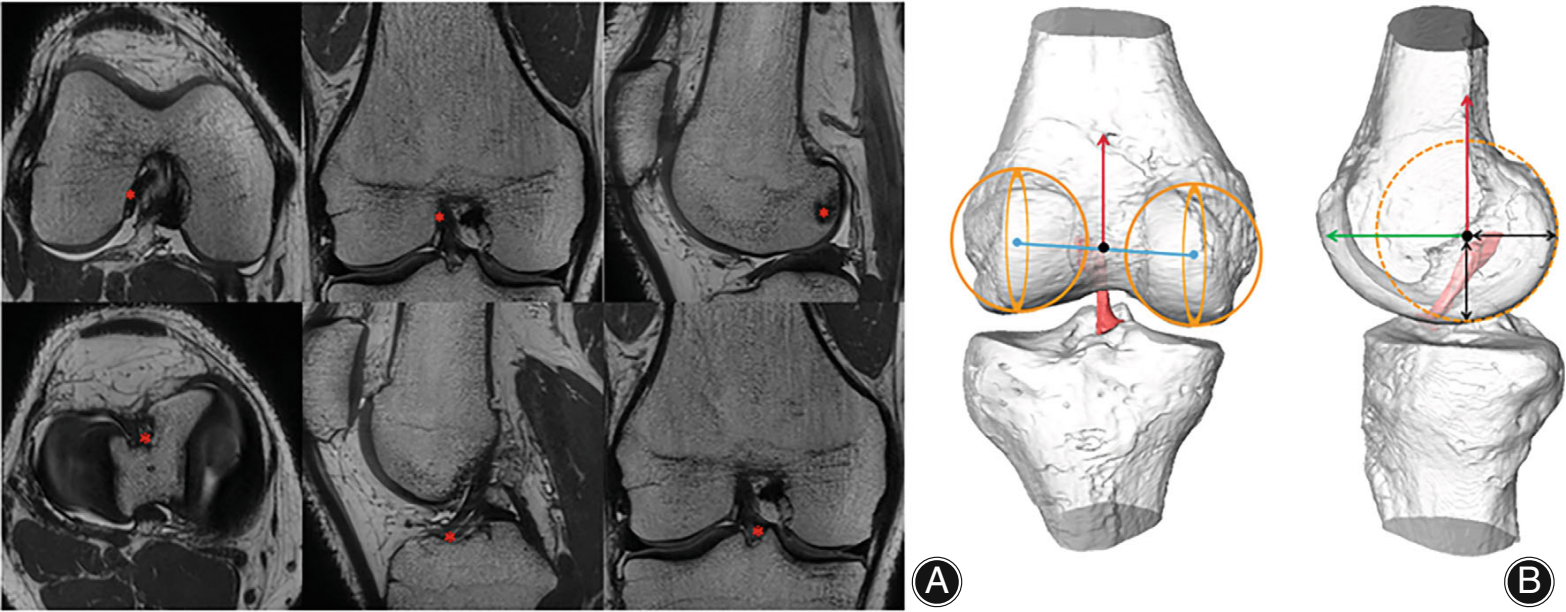
(A) Proton density 3D fast spin‐echo volume sequences (PD space) were applied to collect high‐resolution volumetric MR images, the ACL footprint position is marked with an asterisk. (B) Three‐dimensional surface models of the femur, tibia and ACL were reconstructed. The anatomic coordinate system of the femur is constructed. The origin is shown with a black circle; the mediolateral axis, connecting the center of the best‐fitted spheres on the posterior articular surface of the medial and lateral condyles, is shown with a blue line. The distal‐proximal is demonstrated with a red arrow and the anterior‐posterior axis, a green arrow. The lateral femoral condyle width, defined as the radius of the best‐fitted sphere in the lateral condyle, is shown with a black arrow. ACL, anterior cruciate ligament; MR, magnetic resonance.

Three‐dimensional (3D) MRI, a method that has been proven to have strong agreement with open cadaveric dissection (considered the gold standard), and can be used interchangeably.^26^, ^27^ All enrolled subjects were scanned by a 3.0‐T MRI system with fully extended knees. Proton density 3D fast spin‐echo volume sequences (Skyra; Siemens, Guangzhou, China) were applied to collect Chinese images (slice thickness: 0.50 mm, voxel size: 0.3 × 0.3 × 0.5 mm) (Figure 1A). A 3.0‐T MR Scanner (Achieva; Philips, Zurich, Switzerland) with proton density turbo spin‐echo SPectral Attenuated Inversion Recovery was applied to collect Caucasian images (slice thickness: 1.00 mm, voxel size 0.12 by 2.74 by 0.12 mm). 3D models of femur and tibia were reconstructed using Amira 6.5 FEI SVG (Thermo Fisher, Stoney Creek, California, USA) according to proven and publicly available methods.^28^, ^29^ With the same software, the ACL footprint areas of the femur and tibia were manually depicted on MRI images (Figure 1A).^30^, ^31^ The subsequent analyses of the 3D models was completed by MATLAB2014.

### Concept of the Distal Femur Coordinate System

A femur anatomical coordinate system (fACS) was developed based on a previously published method.^32^ The posterior articular surfaces of the medial and lateral condyles were best fitted and generated two fitted spheres, respectively. The best fitted spherical radius of the lateral condyle was defined as the width of the lateral femur condyle. The mediolateral (M/L) axis was parallel to the line connecting the center of these two fitted spheres. The fACS's M/L axis shared a common line with the flexion–extension axis (FEA) of the knee,^33^, ^34^ because several studies have reported that the cylindrical axis was consistent with the FEA of the knee. The anteroposterior (A/P) axis was perpendicular to both the M/L axis and the best‐fitted cylinder of distal femoral shaft. The distal‐proximal (D/P) axis of the fACS was perpendicular to both the ML axis and the AP axis. The coordinate system originated from the midpoint of the line connecting two condylar fitted spheres (Figure 1B).^35^

### Description of Measurement Indicators

**FIGURE 2:**
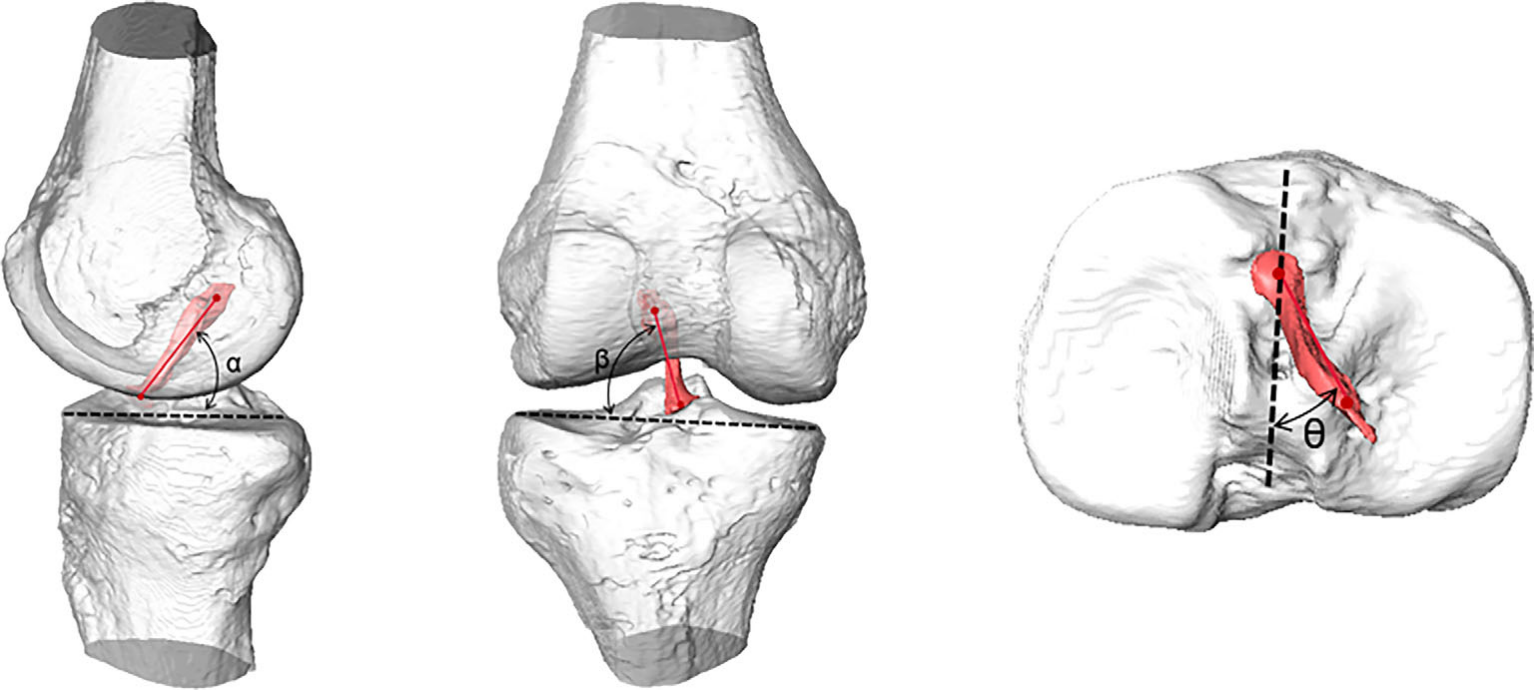
The ACL orientation at full extension position is quantified. The coronal plane elevation angle (β) was defined as the angle between the ACL projected onto the coronal plane of the tibia and the mediolateral axis of the coordinate system. The sagittal plane elevation angle (α) was defined as the angle between the ACL projected onto the sagittal plane of the tibia and the anterior‐posterior axis. The transverse plane deviation angle (θ) was defined as the angle between the projection of the ACL on the tibial plateau and the anterior‐posterior axis. ACL, anterior cruciate ligament.

The centers of femoral and tibial ACL footprints were defined as the barycenter of the footprint surface areas. The locations of the femoral ACL footprint were represented by its distance from the origin in the A/P and D/P directions. These distance values were measured in the software described above, generating values in millimeters and keeping only one decimal place. The normalized location of the femoral footprint was determined by dividing the length in the A/P and D/P directions by the best fitted spherical radius of the lateral condyle, respectively. Positive values represented a more anterior and proximal position relative to the origin of the fACS (or FEA). The ACL vector was defined as the line connecting the centers of the femoral and tibial ACL footprint. The orientation of the ACL was described with the angles of sagittal plane elevation, coronal plane elevation, and transverse plane deviation angles (Figure 2). Sagittal plane elevation (*α*) was defined as the angle between the ACL vector projected onto the sagittal plane of the tibia and the tibial AP axis.^36^ Coronal plane elevation (*β*) was defined as the angle between the ACL vector projected onto the coronal plane of the tibia and the tibial ML axis.^37^ Transverse plane deviation (*θ*) was defined as the angle between the projection of the ACL on the tibial plateau and the tibial AP axis.^38^

### Statistical Analysis

G*Power version 3.1 (Franz Faul, University of Kiel, Germany) was used in the post‐hoc power analysis for the statistical power (1 − *β*) estimation, with a medium effect size and *α* = 0.05. Descriptive statistics described all continuous variables regarding mean, standard deviation, and range, while the categorical data on frequency and percentage. The Kolmogorov–Smirnov test was conducted to inspect the sample normality. If the sample fit a normal distribution, we used the two‐sided *t*‐test. Otherwise, Wilcoxon's signed‐rank test would be required for non‐parametric statistics. ACL footprints were depicted on MRI images by two experienced orthopedists (P.L. and H.S.) and repeated twice independently by each orthopedist. In order to determine the repeatability of current study, the intra and interobserver reliability of these parameters was assessed based on single‐measure intraclass correlation coefficients (ICCs) and two independent blinded observers (D.Z. and L.Z.). Multiple regression analysis was used to study whether there was a quantitative correlation between the ACL orientation and the ACL femoral footprint position under the premise of controlling gender, race and other factors, and to provide possible regression models and parameter values. Ultimately, SPSS software (version 23, IBM, Chicago, IL, USA) was used to perform all data analyses. The result with a *p* value <0.05 was considered having statistical significance.

## Results

### Power Analysis and Reliability Analysis

Statistical power to identify differences in ACL femoral attachment in each group were 95% both for the A/P and D/P locations. The intra and interobserver ICC of all the groups fell in the range of 0.82 to 0.93.

### ACL Femoral Footprint

**FIGURE 3:**
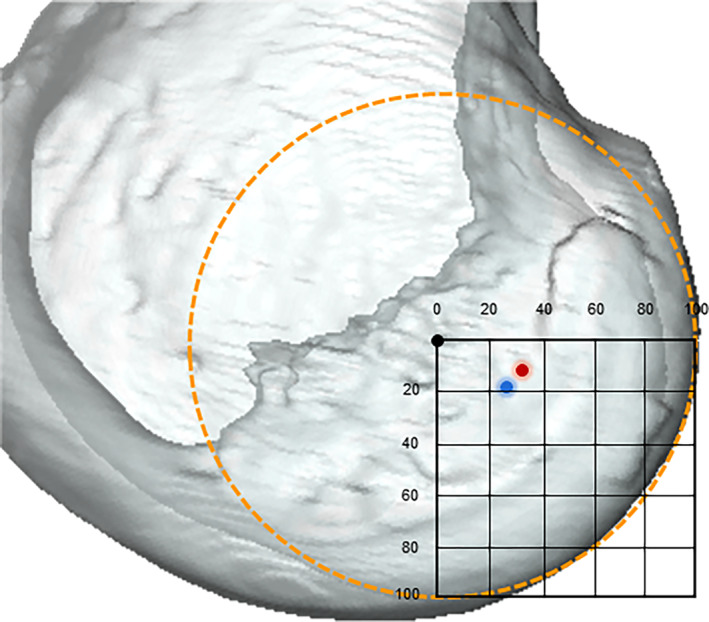
The normalized femoral ACL footprint position (the centroid of the footprint surface area) of the Chinese group (red circle) and Caucasian group (blue circle) in the anterior‐posterior and distal‐proximal direction are demonstrated. Chinese group, with a mean difference of an 6.1% more posterior and 5.28% more proximal position from the FEA. ACL, anterior cruciate ligament.

**FIGURE 4:**
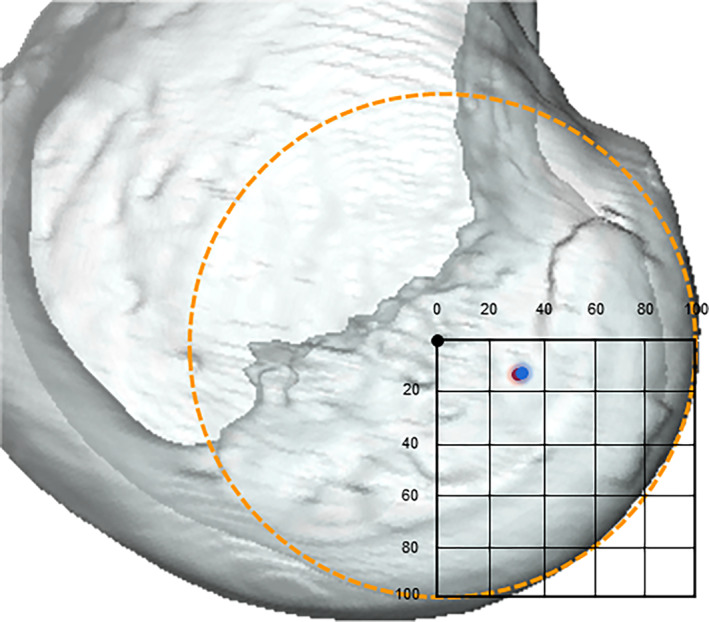
The normalized femoral ACL footprint position (the centroid of the footprint surface area) of the Chinese male group (blue circle) and female group (red circle) in the anterior‐posterior and distal‐proximal direction are demonstrated. The Chinese male had a mean 2.65% more posterior position from the FEA as compared with the Chinese female. ACL, anterior cruciate ligament.

**FIGURE 5:**
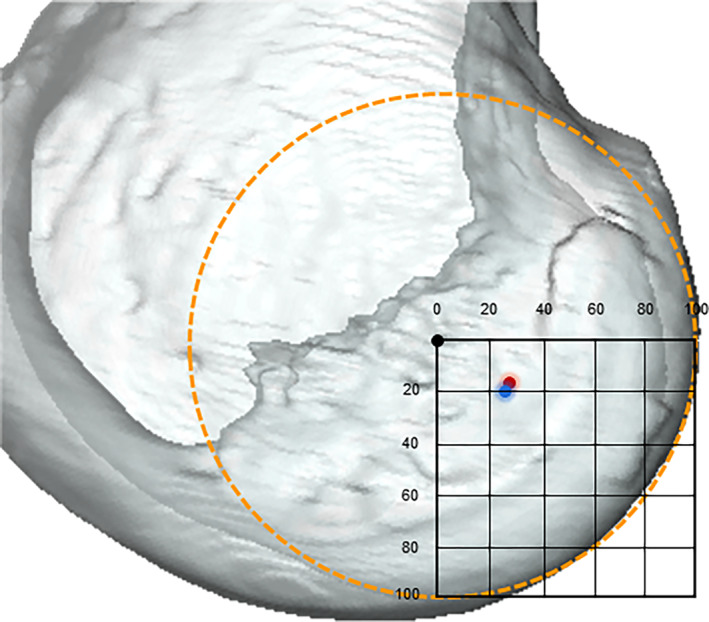
The normalized femoral ACL footprint position (the centroid of the footprint surface area) of the Caucasian male group (blue circle) and female group (red circle) in the anterior‐posterior and distal‐proximal direction are demonstrated. No gender‐specific difference was found in the normalized femur footprint locations with the Caucasian group. ACL, anterior cruciate ligament.

The average A/P position of the ACL femoral footprint was −6.6 ± 1.6 mm in the Chinese group and −5.1 ± 2.3 mm in the Caucasian group (*p* < 0.001) (Figure 3). The normalized A/P position of the ACL femoral footprint was located at a mean of 32.1 ± 6.7% and 26.0 ± 11.9% posterior to the FEA of the knee in the Chinese and Caucasian groups (*p* < 0.001) (Table 2). The average D/P position of the femoral footprint was −2.8 ± 2.4 mm in the Chinese group and −3.9 ± 2.0 mm in the Caucasian group (*p* = 0.001). The normalized D/P position of the ACL femoral footprint was located at a mean of 13.8 ± 11.2% and 19.1 ± 9.8% distal to the FEA of the knee in the Chinese and Caucasian groups (*p* < 0.001) (Table 2). The average A/P and D/P positions of ACL femoral footprint in Chinese males were −7.0 ± 1.6 mm (32.8 ± 6.8%) and −2.8 ± 2.7 mm (13.3 ± 12.6%) (Figure 4); for the Chinese females, the result was −5.7 ± 0.9 mm (30.2 ± 5.1%) and −2.9 ± 1.3 mm (15.2 ± 7.0%) (Table 3). In the Caucasian males, the femur attachment was located at −5.2 ± 2.5 mm (25.4 ± 13.0%) and −4.2 ± 2.1 mm (19.9 ± 9.5%) in A/P and D/P directions of the lateral condyle on average, and for the Caucasian females, the result was −4.9 ± 1.7 mm (27.2 ± 8.9%) in the A/P direction and −3.1 ± 1.4 mm (17.2 ± 6.7%) in the D/P direction (Table 4) (Figure 5).

**TABLE 2:** ACL femur footprint location and orientation between Chinese and Caucasians

Parameter	Chinese (*n* = 90)	Caucasian (*n* = 90)	Statistic value (*t*)	*p*‐value
A/P (mm)	−6.6 ± 1.6	−5.1 ± 2.3	−5.008	<0.001
D/P (mm)	−2.8 ± 2.4	−3.9 ± 2.0	−3.351	0.001
Normalized A/P (%)	32.1 ± 6.7	26.0 ± 11.9	4.243	<0.001
Normalized D/P (%)	13.8 ± 11.2	19.1 ± 9.8	−3.389	<0.001
Alpha (°)	51.8 ± 5.7	51.9 ± 6.1	−0.114	0.921
Beta (°)	66.0 ± 6.3	66.5 ± 7.7	−‐1.290	0.167
Theta (°)	30.5 ± 6.2	29.0 ± 9.4	−1.011	0.251

Abbreviation: ACL, anterior cruciate ligament; A/P anteroposterior; D/P distal‐proximal, Alpha the sagittal draft angle, Beta the coronal draft angle, Theta the transverse draft angle, statistic values (t).

* 0.01 ≤ *p* < 0.05

** 0.001 ≤ *p* < 0.01

*** *p* < 0.001.

**TABLE 3:** ACL femur footprint location and orientation between Chinese males and females

Parameter	Male (*n* = 60)	Female (*n* = 30)	Statistic value (*t*)	*p*‐value
A/P (mm)	−7.0 ± 1.6	−5.7 ± 0.9	6.746	<0.001
D/P (mm)	−2.8 ± 2.7	−2.9 ± 1.3	−0.318	0.869
Normalized A/P (%)	32.8 ± 6.8	30.2 ± 5.1	2.912	0.047
Normalized D/P (%)	13.3 ± 12.6	15.2 ± 7.0	−0.924	0.375
Alpha (°)	53.0 ± 5.6	49.0 ± 4.8	5.161	0.001
Beta (°)	65.8 ± 7.6	63.1 ± 7.1	1.600	0.110
Theta (°)	30.6 ± 6.4	30.2 ± 5.9	0.487	0.799

Abbreviations: ACL, anterior cruciate ligament; A/P, anteroposterior; D/P, distal‐proximal, Alpha the sagittal draft angle, Beta the coronal draft angle, Theta the transverse draft angle, statistic values (*t*).

* 0.01 ≤ *p* < 0.05

** 0.001 ≤ *p* < 0.01

*** *p* < 0.001.

**TABLE 4:** ACL femur footprint location and orientation between Caucasian males and females

Parameter	Male (*n* = 60)	Female (*n* = 30)	Statistic value (*t*)	*p*‐value
A/P (mm)	−5.2 ± 2.5	−4.9 ± 1.7	0.646	0.554
D/P (mm)	−4.2 ± 2.1	−3.1 ± 1.4	3.280	0.010*
Normalized A/P (%)	25.4 ± 13.0	27.2 ± 8.9	−0.709	0.488
Normalized D/P (%)	19.9 ± 9.5	17.2 ± 6.7	1.435	0.151
Alpha (°)	53.3 ± 6.3	48.6 ± 4.2	5.911	<0.001
Beta (°)	66.7 ± 7.6	66.1 ± 8.1	0.563	0.754
Theta (°)	30.0 ± 9.6	26.7 ± 8.5	1.451	0.130

Abbreviations: ACL, anterior cruciate ligament; A/P, anteroposterior; D/P, distal‐proximal, Alpha the sagittal draft angle, Beta the coronal draft angle, Theta the transverse draft angle, statistic values (t).

* 0.01 ≤ *p* < 0.05

** 0.001 ≤ *p* < 0.01

*** *p* < 0.001.

### Orientation of the ACL

The mean angle of sagittal plane elevation was 51.8° ± 5.7° in the Chinese group and 51.9° ± 6.1° in the Caucasian group (*p* = 0.921). The mean angle of coronal plane elevation was 66.0° ± 6.3° and 66.5° ± 7.7°, respectively (*p* = 0.167), and the mean angle of transverse plane deviation was 30.5° ± 6.2° and 29.0° ± 9.4°, respectively (*p* = 0.251) (Table 2). Similarly, the mean angle of sagittal plane elevation was 53.0° ± 5.6° in the Chinese males and 49.0° ± 4.8° in the Chinese females (*p* = 0.001). The mean angle of coronal plane elevation was 65.8° ± 7.6° and 63.1° ± 7.1°, respectively (*p* = 0.110), and the mean angle of transverse plane deviation was 30.6° ± 6.4° and 30.2° ± 5.9°, respectively (*p* = 0.799) (Table 3). And the result for the Caucasian males and females was 53.3° ± 6.3° and 48.6° ± 4.2° in the sagittal plane, respectively (*p* < 0.001), and 66.7° ± 7.6° and 66.1° ± 8.1° in the coronal plane, respectively (*p* = 0.754), and 30.0° ± 9.6° and 26.7° ± 8.5° in the transverse plane, respectively (*p* = 0.130) (Table 4).

### Multiple Regression Analysis

**FIGURE 6:**
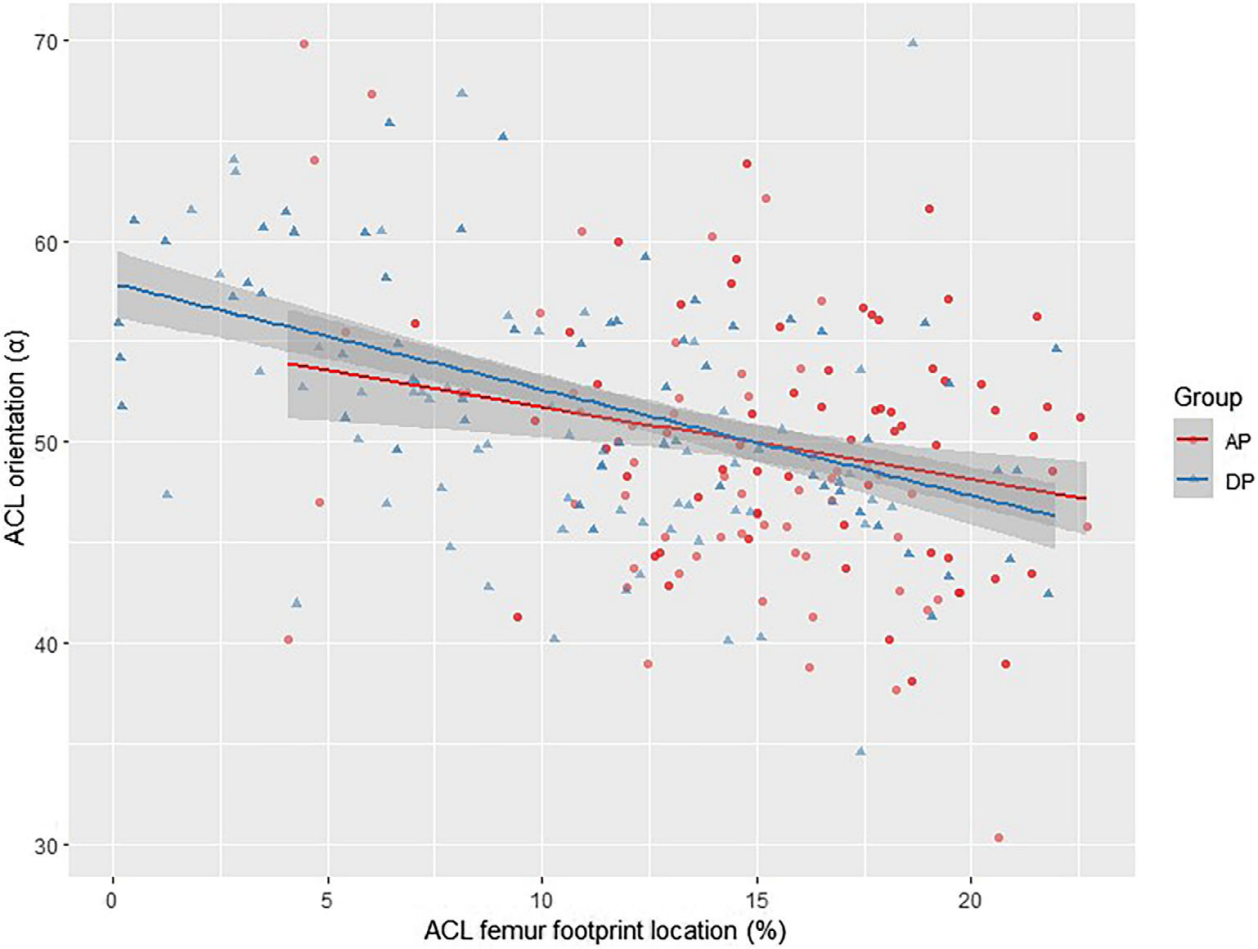
Scatter‐plot of the correlation between location and transverse orientation.

**FIGURE 7:**
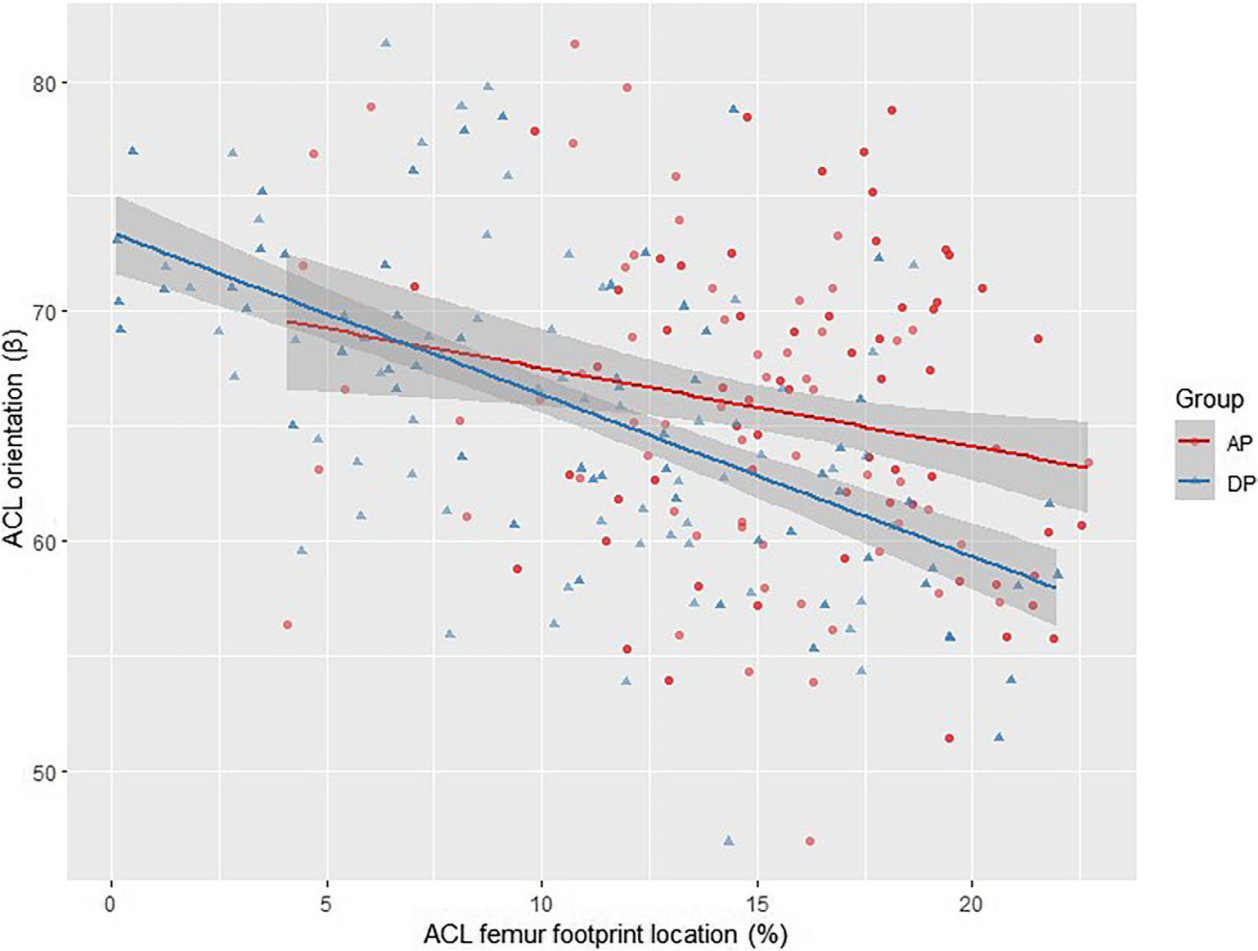
Scatter‐plot of the correlation between location and sagittal orientation.

**FIGURE 8:**
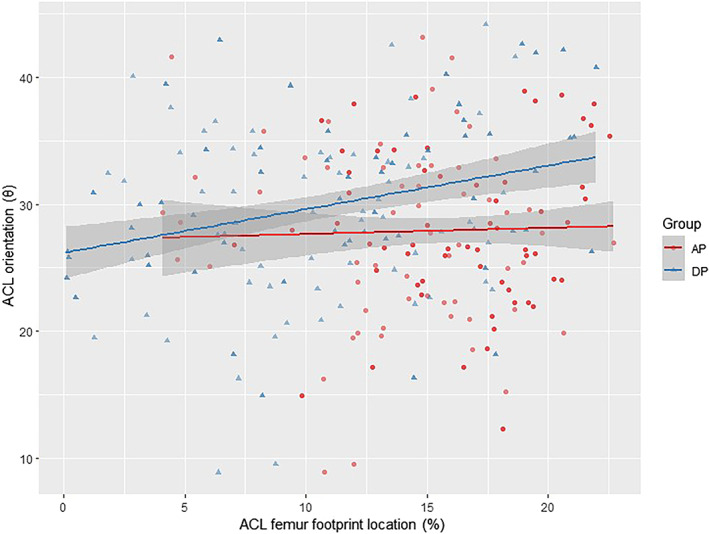
Scatter‐plot of the correlation between location and coronal orientation.

There were multiple linear correlations between the ACL orientation and the femoral footprint location. There was a negative linear correlation between sagittal angle and the femoral footprint position in the A/P and D/P directions (*p* = 0.004, *p* = 1.44e−09). For every 1% (0.40 mm) increase in A/P and D/P values, sagittal angle decreased by about 0.12° and 0.24°, respectively. There was also a negative linear correlation between coronal angle and the ACL femoral footprint position in the A/P and D/P directions (*p* = 0.045, *p* = 1.51e−09). For every 1% (0.40 mm) increase in A/P and D/P values, coronal angle decreased by about 0.10° and 0.30°, respectively. There was a positive linear correlation between the transverse angle and the ACL femoral footprint position in the D/P direction (*p* = 0.018). For every 1% (0.40 mm) increase in the D/P value, the transverse angle increased by about 0.14° (Table 5). Scatter plots were used to visualize the regression correlations (Figures 6, 7, 8). The 95% CI was *β* ± std., and the Regression F‐statistic was 164 (*p* < 0.001).

**TABLE 5:** Multiple regression analysis between ACL orientation and location

The ACL orientation (Dependent variable)	Anterior cruciate Ligament footprint location (independent variable)	Estimate	Std. Error	*t* value	*p* value	*R* ^2^
The sagittal draft angle (α)	Normalized A/P (%)	−0.11919	0.04023	−2.962	0.004**	0.702
	Normalized D/P (%)	−0.24158	0.03762	−6.422	1.44e‐09***	
The coronal draft angle (β)	Normalized A/P (%)	−0.10330	0.05124	−2.016	0.045*	0.674
	Normalized D/P (%)	−0.30721	0.04790	−6.413	1.51e‐09***	
The transverse draft angle (θ)	Normalized A/P (%)	0.004365	0.063776	0.068	0.946	0.578
	Normalized D/P (%)	0.142199	0.059627	2.385	0.018*	

Abbreviations: ACL, anterior cruciate ligament; A/P, anteroposterior; D/P, distal‐proximal, *α*, the sagittal draft angle, *β*, the coronal draft angle, *θ*, the transverse draft angle.

* 0.01 < *p* < 0.05

** 0.001 < *p* < 0.01

*** *p* < 0.001.

## Discussion

### Significant Race and Gender Difference in Anterior Cruciate Ligament Femoral Footprint Location and Orientation

In this study, 3D‐MRI images were used to find out whether there were significant differences between race and gender in the ACL femoral attachment and the ACL orientation, and to analyze the relationship between ACL orientation and ACL femoral attachment. There was a significant race‐specific difference in femoral footprint locations. Compared with the Caucasian group, the Chinese group had a mean difference of an 1.5 mm (6.1%) more posterior and 1.1 mm (5.3%) more proximal position from the FEA. The Chinese male had a mean 1.3 mm (2.6%) more posterior position from the FEA as compared with the Chinese female, but no gender‐specific difference was found about the normalized femur footprint locations in the Caucasian group. It was also demonstrated that a significant gender‐specific difference existed in the ACL orientation, with the sagittal plane elevation angle being 4–5° higher in males than in females, regardless of races. Furthermore, the coronal angle and sagittal angle were negatively correlated with the A/P and D/P values. For every 1% (0.40 mm) increase in A/P and D/P values, the sagittal angle decreased by about 0.12° and 0.24°, respectively; the coronal angle decreased by about 0.10° and 0.30°, respectively. However, the transverse angle was positively correlated with the D/P value. For every 1% (0.40 mm) increase in D/P value, the transverse angle increased by about 0.14°.

### Femur Footprint Location and ACL Orientation

**FIGURE 9:**
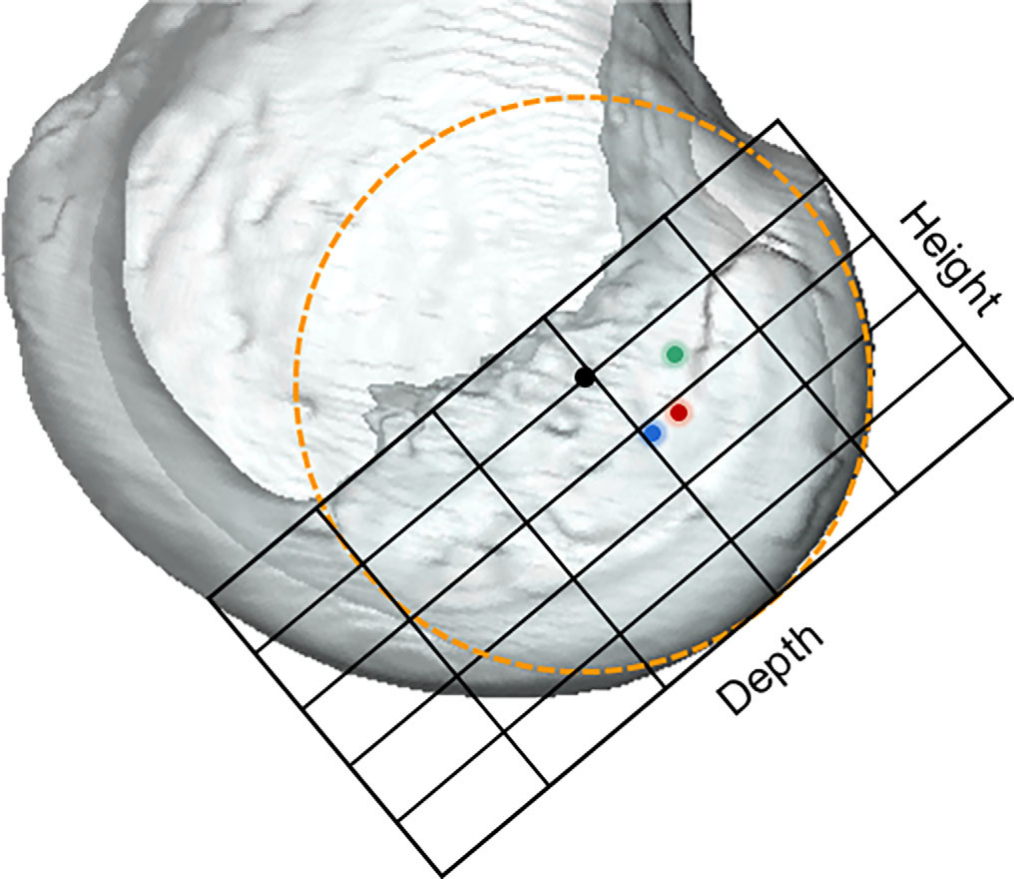
The normalized femoral ACL footprint position in the anterior‐posterior and distal‐proximal direction in Chinese group (red circle); and Caucasian group (blue circle) is demonstrated. The mean position of the femoral ACL footprint reported in the literature is also shown (green circle) based on the quadrant method. The black circle indicates the origin (or flexion extension axis) of the femoral coordinate system. ACL, anterior cruciate ligament.

Individualized femoral tunnel positioning was still a research hotspot in anatomical single‐bundle ACL reconstruction (Figure 9). Yamamoto *et al*.^14^ reported a study of cadavers with an average age of 48.6 years, in which the femur attachment was located at 29% of the height measured from the Blumensaat line and 27% of the depth measured from the posterior margin of the lateral femoral condyle on average, respectively. Steckel *et al*.^33^ reported in their two‐dimensional radiographs study that the femur attachment was located at 27.5% in the A/P direction and 26.9% in the D/P direction on average. Edwards *et al*.^34^ reported a study with 22 elderly cadaver knees that the femur attachment center was located at 38.0% of the height and 41.5% of the depth based on the Amis' circle.^39^ In addition, Reid *et al*.^36^ noted that the mean angle of inclination was 46.9° ± 4.9° in the sagittal plane and 74.3° ± 4.8° in the coronal plane based on MRI of 188 patients with intact ACLs. Freddie *et al*.^40^, ^41^ evaluated the native ACL sagittal angle was 49.9° ± 2.8°, and the femoral footprint was located at 27.5 ± 4.4% in the A/P direction and 38.5 ± 9.2% in the D/P direction. The femur footprint was located at 29.0% in the A/P direction and 16.5% in the D/P direction on average in the present study. (Tables 2, 3, 4). However, the current study also reported additional findings, namely, the femur footprint location was 1.5 mm (6.1%) more posterior and 1.1 mm (5.3%) more proximal in the Chinese group, especially, the Chinese males had a mean 1.3 mm (2.6%) more posterior position from the FEA as compared with the Chinese female, and the sagittal plane elevation angle was 4–5° higher in men than in women, regardless of race.

### Previously Reported Grafts Generated by AM and TT Techniques Were Compared with Native ACLs in the Present Study

In addition, anteromedial (AM) portal and transtibial (TT) drilling techniques were two common options for drilling femoral tunnels. It was reported that there were significant differences in the position, orientation and length of the femoral tunnels obtained by these two approaches.^42^, ^43^, ^44^ Based on several studies,^45^, ^46^, ^47^ the mean sagittal graft angle was 51.8° and 63.3° in the AM and TT groups, respectively. Among these studies, Guler *et al*.^38^ additionally reported that according to the quadrant method the graft insertion was located at 31.33% with AM technology and 29.53% with TT technology in the A/P direction. In a comparative study of X‐ray plain radiographs obtained from 404 patients undergoing ACL reconstruction, Gabr *et al*.^39^ found the mean femoral tunnel position along the Blumensaat's line relating to the posterior femoral cortex was 16% in the AM group *versus* 22% in the TT group; the mean graft coronal inclination angle was 31.9° in the AM group and 22° in the TT group. In the present study, our results were similar to theirs (Tables 2, 3, 4). It was worth noting that the mean sagittal orientation of the native ACL (51.85°in this study) was more similar to the graft angle of the AM technique (51.80° from the literature). And there was no race‐specific difference in the ACL orientation, but males have a sagittal plane elevation about 4–5° higher than females in different race groups, respectively (Tables 2, 3, 4, 5). The results of the current study suggested that surgeons should pay special attention to the race‐specific differences in the femoral ACL footprint and gender‐specific in the orientation of ACL when individually customizing bone tunnels for patients.

### The Anatomical Characteristics of Native ACL Might Have Implications for ACL Injury and Surgical Reconstruction

In a cadaveric study comparing anterior and rotational stability between conventional single‐bundle ACL reconstruction and anatomical fibers, Brophy *et al*.^40^ confirmed that a more posteriorly located femoral graft (posterolateral placement) could be more susceptible to apparent strain in extension or internal rotation. This might also be attributable to the high failure rate in the posterolateral bundle of the double‐bundle reconstruction.^41^ Further studies showed that the femoral graft position obtained by the TT drilling technique was more proximal and more anterior with a larger graft inclination angle, leading to abnormal knee kinematics and a higher extension–flexion pattern compared to the native ACL, while the latter could result in residual rotational instability, flexion restriction, potential graft impingement, or even graft failure.^48^, ^49^, ^50^, ^51^, ^52^ In the Chinese group, a more posteriorly and proximally located femoral ACL footprint might increase the risk of ACL injury, and a larger ACL inclination angle in males than in females might be correlated with a higher risk of ACL injury in non‐athlete males,^53^, ^54^ owing to the altered kinematics and loading mechanics during a noncontact injury. However, the report on ACL injury rate in Chinese non‐athletes was still limited. The results of the relationship between the ACL footprint location and the orientation might be useful to evaluate the current tunnel positions and determine the preoperative planning. In particular, for patients with graft failure, abnormal graft inclination and postoperative complications, our findings may provide a reference for surgeons to develop the revision operative strategy.

### Limitations

This study should be interpreted in consideration of its limitations. First, MRI‐based 3D models were used to identify the femoral and tibial footprints. Even though cadaveric studies were considered as the gold standard, the 3D‐MRI has been proven to be an accurate and reliable imaging method^27^, ^55^, ^56^ in that setting and larger populations could be included. Moreover, since the subjects in this study were Chinese and Caucasian with intact ACLs, further studies should include patients with ACL injury from different ethnic groups.

## Conclusion

The significant race‐ and gender‐specific differences in the femoral footprint and orientation of the ACL should be taken in consideration during anatomic single‐bundle ACL reconstruction. Furthermore, the quantitative relationship between ACL orientation and ACL footprint location might be useful for surgeons to evaluate the current tunnel positions, determine the preoperative planning, and develop the revision operative strategy.

## Funding Information

This study was supported by the National Natural Science Foundation of China (Grant Numbers 81871808), Military Project of Application Fundamentals (Grant Number CLB21J021, 20QNPY084, CLB18J037 and 21FYFH06), Pudong New Area Science & Technology Development Fund [210H1147900], National Natural Science Foundation of China [31972924], “Science and Technology Innovation Action Plan” of Science and Technology Commission of Shanghai Municipality [22S31906000, 21DZ2208200, 23S31901000], the Fundamental Research Funds for the Central Universities [AF0820060].

## Conflict of Interest Statement

The authors declare there is no conflict of interest.

## Ethics Statement

This study was approved by the ethics committee of Chinese PLA Southern Theater Command General Hospital (2019‐No.10) and the ethics committee of Northeast and Central Switzerland (2018‐01410).

## Author Contributions

Design: Lihang Zhang, Tsung‐Yuan Tsai, Pingyue Li. Data acquisition: Tianwen Huang, Changzhao Li, Diyang Zou. Analysis and interpretation of data: Dimitris Dimitriou, Xing Xing.
